# Effect of chemotherapy on cancer specific mortality in female locally advanced urethral cancer

**DOI:** 10.1007/s00345-025-05545-0

**Published:** 2025-05-04

**Authors:** Quynh Chi Le, Natali Rodriguez Peñaranda, Andrea Marmiroli, Francesco di Bello, Mattia Longoni, Fabian Falkenbach, Calogero Catanzaro, Michele Nicolazzini, Zhe Tian, Jordan A. Goyal, Carolin Siech, Cristina Cano Garcia, Fred Saad, Riccardo Schiavina, Salvatore Micali, Stefano Puliatti, Ottavio De Cobelli, Alberto Briganti, Markus Graefen, Carlotta Palumbo, Alessandro Volpe, Luis A. Kluth, Felix K-H. Chun, Pierre I. Karakiewicz

**Affiliations:** 1 Cancer Prognostics and Health Outcomes Unit, Division of Urology, University of Montréal Health Center, Montréal, Québec Canada; 2 Department of Urology, Goethe University Frankfurt, University Hospital, Frankfurt am Main, Germany; 3 Department of Urology, University of Bologna, St. Orsola-Malpighi Hospital, Bologna, Italy; 4 Department of Urology, AOU di Modena, University of Modena and Reggio Emilia, Modena, Italy; 5 Department of Urology, IEO European Institute of Oncology, IRCCS, Via Ripamonti 435, Milan, Italy; 6 Università degli Studi di Milano, Milan, Italy; 7 Vita-Salute San Raffaele University, Milan, Italy; 8 Division of Experimental Oncology, Unit of Urology, URI, Urological Research Institute, IRCCS San Raffaele Scientific Institute, Milan, Italy; 9 Martini-Klinik Prostate Cancer Center, University Hospital Hamburg-Eppendorf, Hamburg, Germany; 10 Division of Urology, Department of Translational Medicine, University of Eastern Piedmont, Maggiore della Carità Hospital, Novara, Italy; 11 Division of Urology, Department of Oncology, University of Turin, Orbassano, Italy; 12 Department of Neurosciences, Science of Reproduction and Odontostomatology, University of Naples Federico II, Naples, Italy

**Keywords:** Locally advanced urethral cancer, Females, Chemotherapy, Cancer specific mortality

## Abstract

**Objective:**

To quantify the effect of chemotherapy (CHT) in locally advanced female primary urethral cancer (fPUC).

**Methods:**

In the Surveillance, Epidemiology and Ends Results (SEER) database (2000–2021), we identified 295 fPUC patients with locally advanced stage treated with local therapy (surgery or radiation or both) with or without CHT. Multivariable Cox regression models addressed cancer specific mortality free survival (CSM). Sample power analyses were computed.

**Results:**

Of 295 fPUC patients, 141 (48%) underwent CHT. CHT rates increased from 40 to 61% (Δ22%) over the study span (2000–2021). Five-year CSM rates of CHT exposed vs. CHT-naïve patients were 58 vs. 43% (Δ15%). In multivariable Cox regression models (age and histology adjusted) CHT independently predicted lower CSM (HR = 0.67, *p* = 0.027). In squamous cell carcinoma (SCC) subgroup, CHT also independently predicted lower CSM (HR = 0.64, *p* = 0.01). In urothelial carcinoma (HR = 0.63, *p* = 0.2) and adenocarcinoma (HR = 0.7, *p* = 0.7) independent predictor status could not be demonstrated. Small sample sizes in urothelial carcinoma subgroup (UC) and adenocarcinoma subgroup (ADK) undermined the power of the analyses to as low as 48% in UC and 46% in ADK, respectively, versus ideal 80% power.

**Conclusion:**

In fPUC patients, CHT independently predicts lower CSM. This effect is generalizable to SCC patients. The same relationship between CHT status and CSM is also operational in UC and ADK subgroups, but limited power undermined confirmation of its’ statistical significance.

## Introduction

Primary urethral cancer (PUC) accounts for less than 1% of urological malignancies. Its’ aged-standardized rate is 1.5 per million in females and 4.3 per million in males in North America [1]. Due to male predominance of PUC [2, 3], most studies address mainly males, for example Gakis et al. (*n* = 154) reported 70% male membership, Wenzel et al. (*n* = 1073) 65% male membership and Sui et al. (*n* = 2137) 60% male membership [4–6]. In consequence, treatment types and cancer specific mortality (CSM) rates in female PUC (fPUC) are relatively unknown. Unfortunately, only three analyses specifically examined PUC in female patients. For example Derksen et al. (1989–2008) described 91 fPUC vs. Peyton et al. (2003–2017) described 39 fPUC vs. Lee et al. (1997–2017) described 32 fPUC. However, none of these studies specifically examined locally advanced fPUC or the association between CHT and CSM.

Within PUC, locally advanced patients are known to harbor suboptimal cancer control outcomes. However, in these individuals, it is possible to improve cancer control outcomes by virtue of optimizing treatment intensity. To the best of our knowledge, no study on locally advanced fPUC was reported so far.

We addressed this knowledge gap with the intent of quantifying the association between CHT and CSM in this locally advanced fPUC. We hypothesize that locally advanced fPUC benefit of CHT as recommended by guideline [1]. Moreover, we postulated that CHT use is associated with lower CSM. To examine these hypotheses, we relied on locally advanced fPUC from within the SEER database (2000–2021).

## Methods

### Data source and study population

The SEER database provides cancer statistics for approximately 47.9% of the United States population [7]. Within the SEER database (2000–2021), we identified fPUC with locally advanced stage (T3-T4, N1-N3, M0) aged ≥ 18 years with histologically confirmed PUC (International Classification of Disease for Oncology [ICD-10] site code 68.0) with follow-up-data. Excluded were all autopsy- or death certificate-only cases, as well as patients with unknown histology, unknown stage, histological subtypes other than urothelial (UC), squamous cell carcinoma (SCC) or adenocarcinoma (ADK) or patients who have not underwent either surgery or radiotherapy or both. Due to the anonymous nature of the SEER database, study-specific Institutional Review Board approval was waived.

### Statistical analyses

**Fig. 1:**
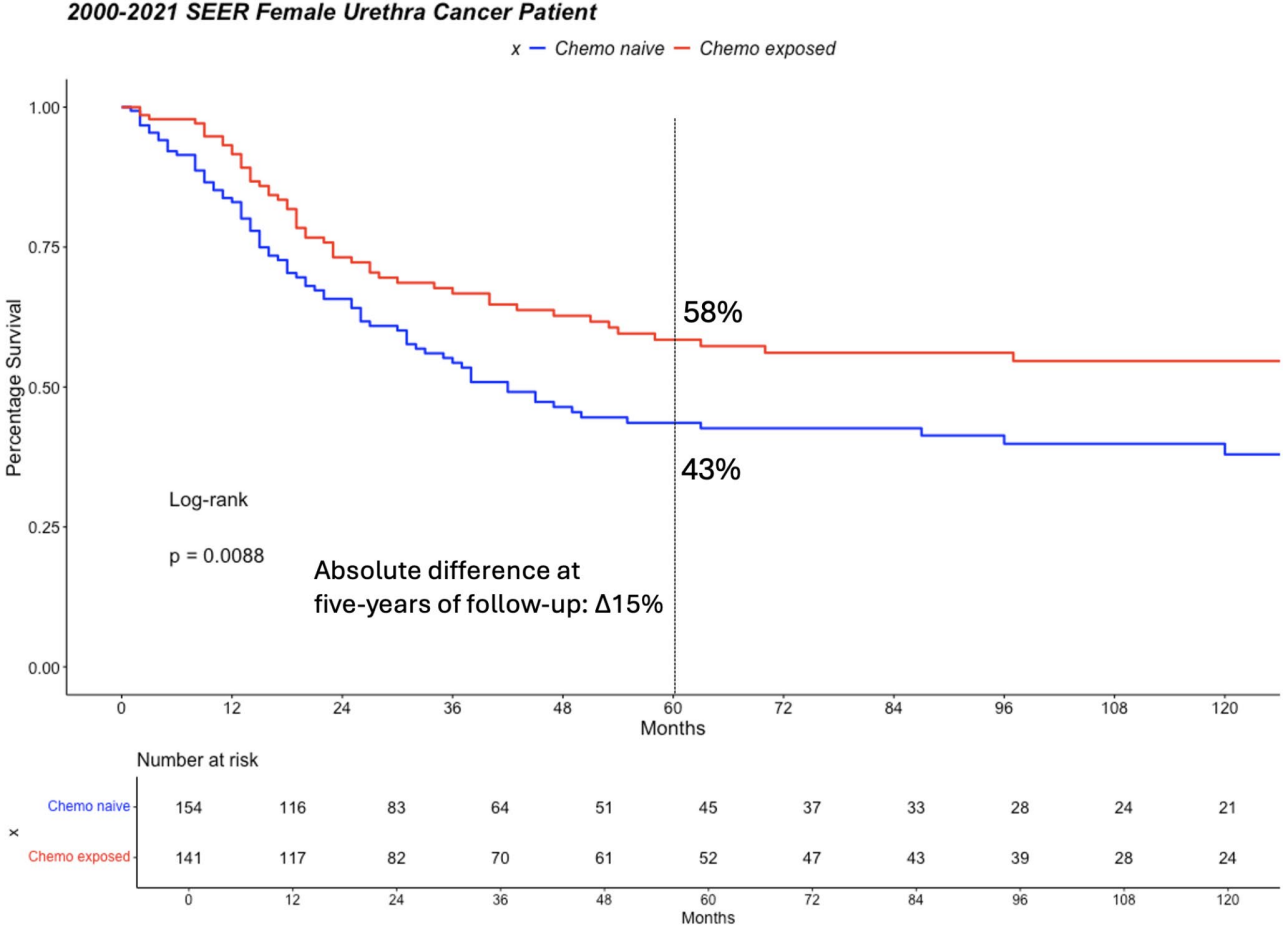
Kaplan-Meier-plots depicting cancer specific mortality-free survival in 295 locally advanced female urethral cancer patients, CHT = Chemotherapy

**Fig. 2:**
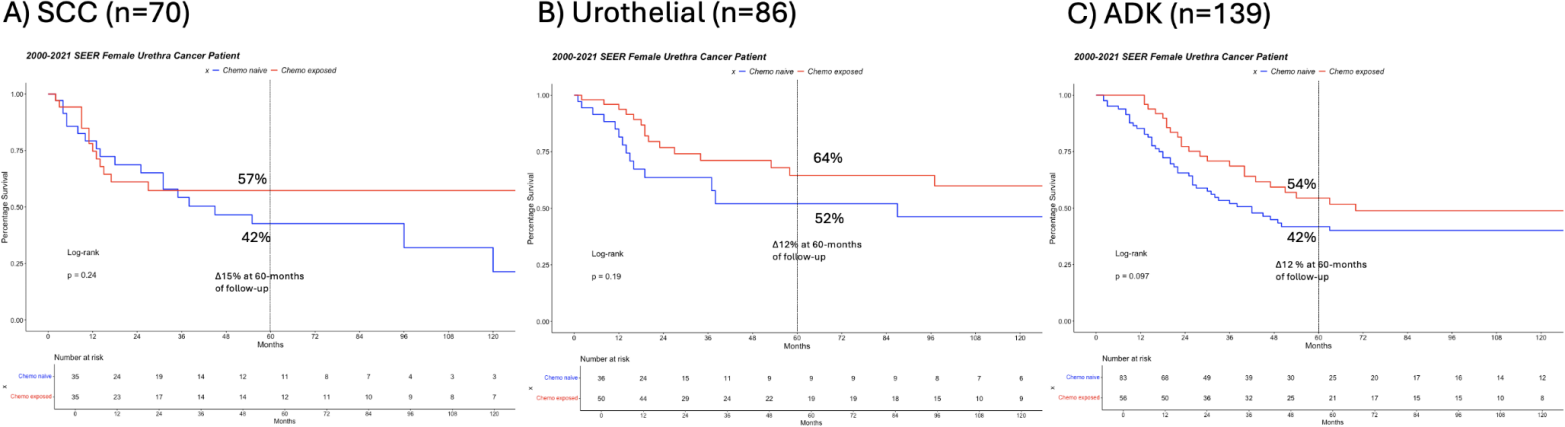
Kaplan Meier curves depicting cancer specific mortality free survival in 295 locally advanced female urethral cancer patients according to histological subtypes, **A**) squamous cell carcinoma, **B**) urothelial carcinoma, and **C**) adenocarcinoma, CHT = Chemotherapy

Baseline characteristics of locally advanced fPUC patients were tabulated (Table 1). Kaplan-Meier plots graphically depicted CSM rates (Figs. 1 and 2). Univariable and multivariable Cox regression models addressed CSM (Tables  2 and 3). Variance inflation factors were addressed to avoid co-linearity within the multivariable Cox regression models. No co-linearity was found in the models. Finally, sample-/ power analyses were computed. Statistical tests were two-sided with a level of significance set at *p* < 0.05. R software environment (R Version 4.4.0, The R Foundation for Statistical Computing, Vienna, Austria) was applied for graphics and statistical computing.

**Table 1:** Descriptive characteristics of 295 female urethral cancer patients with locally advanced stages according to receipt of chemotherapy

Characteristic	Overall *N* = 295^1^	Chemotherapy received, *n* = 141 (48%)^1^	Chemotherapy naive, *n* = 154 (52%)^1^
**Age**	66 (58, 74)	65 (59, 74)	66 (58, 75)
**Follow-Up**	31 (13, 87)	34 (15, 98)	26 (12, 69)
**Histology**			
Urethral	86 (29%)	50 (35%)	36 (23%)
Squamous cell	70 (24%)	35 (25%)	35 (23%)
Adenocarcinoma	139 (47%)	56 (40%)	83 (54%)
**T-Stage**			
T1	17 (5.8%)	15 (10%)	3 (1.9%)
T2	55 (19%)	25 (18%)	30 (19%)
T3	198 (67%)	88 (62%)	110 (71%)
T4	20 (7%)	10 (7%)	10 (7%)
TX	4 (2%)	3 (2%)	1 (1%)
**N-Stage**			
N0	176 (60%)	61 (43%)	115 (75%)
N1	44 (15%)	30 (21%)	14 (9%)
N2	63 (21%)	45 (32%)	18 (12%)
NX	12 (4%)	5 (4%)	7 (5%)

1Median (Q1, Q3); n (%)

**Table 2:** Univariable and multivariable Cox analyses in the overall cohort of 295 female urethral cancer patients with locally advanced stage

	Univariable Cox Analysis			Multivariable Cox Analysis *		
	**HR** ^**1**^	**95% CI** ^**1**^	***p*** **-value**	**HR** ^**1**^	**95% CI** ^**1**^	***p*** **-value**
CHT exposure	0.631	0.5, 0.9	**0.009**	0.670	0.5, 1.0	**0.027**

^1^HR = Hazard Ratio, CI = Confidence Interval, CT = Chemotherapy

**Table 3:** Univariable and multivariable Cox analyses according to histological subtype

SCC				
Univariable Cox Analysis			Multivariable Cox Analysis *	
	HR^1^ (95% CI^1^)	*p*-value	HR^1^	*p*-value
CHT-exposure	**0.63 (**0.4, 0.9)	**0.009**	**0.64 (0.4**,** 0.9)**	**0.01**

**UC**				
**Univariable Cox Analysis**			Multivariable Cox Analysis *	
	**HR** ^**1**^ **(95% CI** ^**1**^ **)**	***p*** **-value**	**HR** ^**1**^	***p*** **-value**
CHT-exposure	0.62 (0.3, 1.3)	0.2	0.63 (0.3, 1.3)	0.2

**ADK**				
**Univariable Cox Analysis**			Multivariable Cox Analysis *	
	**HR** ^**1**^ **(95% CI** ^**1**^ **)**	***p*** **-value**	**HR** ^**1**^	***p*** **-value**
CHT-exposure	**0.7 (**0.5, 1.1)	**0.1**	**0.7 (0.4**,** 1.0)**	**0.7**

^1^HR = Hazard Ratio, CI = Confidence Interval, *adjusted for age

Abbreviations: SCC = squamous cell carcinoma, CHT = Chemotherapy, UC = urothelial carcinoma, CHT = Chemotherapy, ADK = Adenocarcioma, CHT = Chemotherapy

## Results

### Descriptive characteristics

In 295 locally advanced fPUC, median age was 65 years in CHT received vs. 66 years in CHT-naïve patients. Follow-up durations were longer in patients exposed to CHT vs. CHT-naïve patients (34 vs. 26 months). Regarding histological subtypes, 86 (29%) had UC, 70 (24%) SCC, 139 (47%) ADK. According to T-stage, patients exposed to CHT harbored more frequently T3/T4 stages than their CHT-naïve counterparts (77% vs. 69%). Similarly, patients exposed to CHT harbored more N1/N2 stages than their CHT-naïve counterparts (53 vs. 21%).

### Rates of chemotherapy over the study span (2000–2021)

**Fig. 3:**
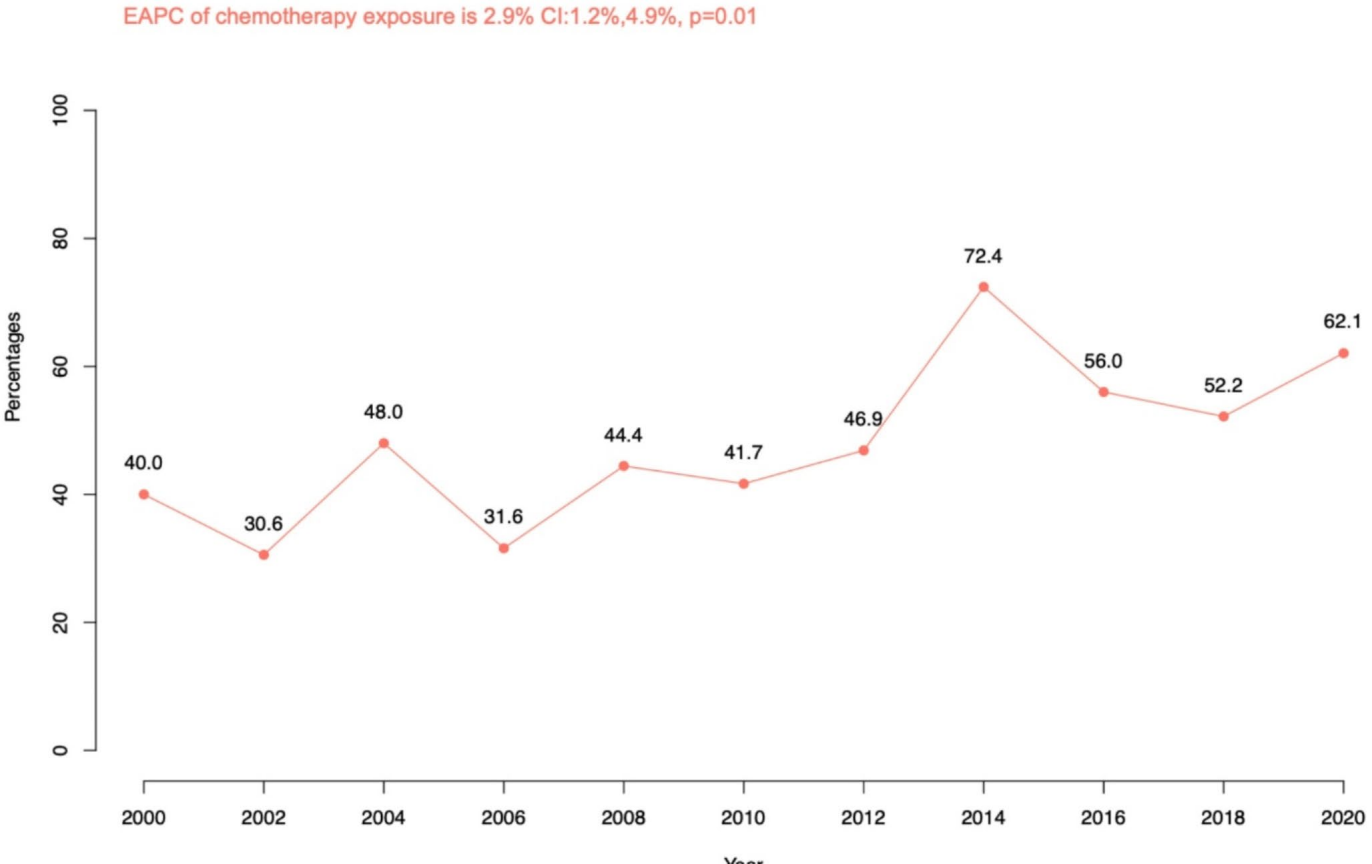
Rates of chemotherapy in locally advanced female urethral cancer patients and estimated annual percentage changes between 2000 and 2021 (years coupled)

Of 295 locally advanced fPUC, 143 (48%) were exposed to CHT (Fig 3). According to study years, the absolute numbers ranged from 14/35 (40%) patients who underwent CHT in 2000–2001 to 18/29 (62%) patients in 2020–2021 The difference over time (Δ22%) corresponded to an estimated annual percentage change of 2.9% (*p* = 0.01).

### Effect of chemotherapy on cancer specific mortality free survival in the overall cohort

Five-year CSM rates in the overall cohort were 58 vs. 43% (Δ15%) in CHT exposed vs. CHT naïve patients, respectively (Fig. 1). These CSM rates yielded a univariable HR = 0.63 (*p* = 0.009). After adjustment for age and histology, a multivariable HR = 0.67 (*p* = 0.027) was recorded.

### Effect of chemotherapy on cancer specific mortality free survival in histological subgroups

In SCC cohort (*n* = 70), 35 patients underwent CHT (50%). Five-year CSM rates were 57% vs. 42% (Δ15%) in CHT exposed vs. CHT naive patients, respectively (Fig. 2A). These CSM rates resulted in a univariable HR = 0.63 (*p* = 0.009). Adjusted for age, a multivariable HR = 0.64 (*p* = 0.01) was recorded.

In UC cohort (*n* = 86), 50 patients underwent CHT (58%). Five-year CSM rates were 64 vs. 52% (Δ12%) in CHT exposed vs. CHT naive patients, respectively (Fig. 2B). These CSM rates resulted in a univariable HR = 0.62 (*p* = 0.2). Adjusted for age, a multivariable HR = 0.63 (*p* = 0.2) was recorded.

In ADK cohort (*n* = 139), 54 patients underwent CHT (40%). Five-years CSM rates were 54 vs. 42% in CHT exposed patients vs. CHT naive patients (Δ12%), respectively (Fig. 2C). These CSM rates resulted in a univariable HR = 0.7 (*p* = 0.1). Adjusted for age, a multivariable HR = 0.7 (*p* = 0.7) was recorded.

### Sample size and sample power calculations in UC and SCC subgroups

Within UC subgroup, 50 vs. 36 patients were CHT exposed vs. CHT naïve. Assuming 36 patients in both arms with 𝛼=0.05, a power of 48% was recorded. In a more favourable scenario where 50 patients would be available in each arm with 𝛼=0.05, a power of 62% was recorded. To detect the observed difference of five-years CSM rates (Δ12%) with ideally 80% power and 𝛼=0.05, at least 213 observations would have been required per arm.

Within ADK subgroup, 56 vs. 83 patients were CHT exposed vs. CHT naïve. Assuming 50 patients in both arms with 𝛼=0.05, a power of 46% was recorded. In a more favourable scenario where 83 patients would be available in each arm with 𝛼=0.05, a power of 62% was recorded. To detect the observed difference of five-years CSM rates (Δ12%) with ideally 80% power and 𝛼=0.05, at least 276 observations would have been required per arm.

## Discussion

Within PUC, locally advanced staged patients may represent the subgroup where cancer control outcomes can be improved according to treatment intensification. However, treatment types and CSM rates in locally advanced fPUC are relatively unknown. We addressed this knowledge gap and made several noteworthy observations.

First, PUC is very rare, especially in females [1, 2]. In the current study, we only identified 295 locally advanced fPUC over a 22 year period (2000–2021). These numbers validate PUC-rarity, especially in females with locally advanced stage [1, 2]. Of three existing studies that exclusively focused on fPUC, the sample sizes ranged from 32 to 91 patients [8–10]. However, none of these studies addressed locally advanced fPUC or the association between CHT and CSM. These knowledge gaps validate the pertinence of the current study that relied on the largest contemporary locally advanced fPUC cohort (*n* = 295) [9–13]. 

Second, median age was 65 in the current study cohort. This is consistent with previous PUC studies that relied on mixed-sex PUC cohorts, where median age ranged from 60 to 66 years [14, 15]. When comparing female subgroups in mixed-sex PUC cohorts with the current study cohort, the proportions of histological subtypes are also similar [3–5, 16]. Previous studies reported ADK as the most common subtype in female subgroups of mixed-sex PUC cohort, followed by UC and SCC [1, 5]. In the current study, most fPUC harbored ADK (47%), followed by UC (29%) and SCC (24%). These observations validate the current study population relative to previous studies that predominantly relied on male patient populations.

Third, we addressed rates of CHT between 2000 and 2021. Out of 295 locally advanced fPUC, 141 patients underwent CHT (48%). The rate of CHT was higher than the ones reported in previous studies. For example, Dayyani et al. (2005–2009) reported on 40 of 140 PUC patients (29%) who underwent CHT. Gakis et al. (1993–2012) reported on 61 of 154 PUC patients (40%) in whom CHT was used [14, 17]. However, previous studies did not allow to separate males from females and ascertain the rates of CHT in female PUC patients alone. Moreover, the annual rates of CHT in locally advanced fPUC in the current study increased from 40% in the initial study years (2000–2001) to 62% in the final study years (2020–2021). This observation is both novel and encouraging and cannot be directly compared to previous studies.

Fourth, we examined the association between CHT exposure and CSM in the overall fPUC cohort. Five-year CSM rates were 58 vs. 43% (Δ15%) in CHT exposed vs. CHT naive patients, respectively (Fig. 3). This resulted in a multivariable HR of 0.67 (*p* = 0.027) where CHT reached independent predictor status for lower CSM. This observation is also novel and encouraging. It validates the benefit of CHT in locally advanced fPUC patients in accordance with guideline recommendations. To the best of our knowledge, the current study represents a first objective validation that is based on strict statistical testing with proven independent predictor status. The observations cannot be directly compared to previous studies, since no previous studies examined locally advanced fPUC.

Fifth, we examined the association between CHT exposure and CSM in specific histological subgroups. In SCC patients, five-year CSM rates were 57 vs. 42% (Δ15%) in CHT-exposed vs. CHT naive patients, respectively. This resulted in a multivariable HR of 0.64 (*p* = 0.01). In UC, five-year CSM rates were 64 vs. 52% (Δ12%) in CHT exposed vs. CHT naive patients, respectively. This resulted in a multivariable HR of 0.63 (*p* = 0.2). In ADK cohort, five-years CSM rates were 54 vs. 42% (Δ12%) in CHT-exposed vs. CHT naive patients, respectively. This resulted in a multivariable HR of 0.7 (*p* = 0.7). Independent predictor status could only be recorded in SCC subgroup. Due to limited sample sizes, independent predictor status for lower CSM could not be recorded in UC and ADK subgroups. Lack of independent predictor status may predominantly be explained by small numbers of observations in those two subgroups, since the effect sizes were virtually the same across all three histological subtypes. Based on the sample sizes at hand, power calculations demonstrated severely limited power in UC (48–62%) and in ADK (46–62%) subgroups relative to ideal power of 80%. In consequence, availability of larger sample sizes might be expected to result in statistically significant differences and possibly independent predictor status of CHT for lower CSM in UC and ADK subgroupd. Unfortunately, it is unlikely that larger samples sizes may be identified in both subgroups.

Taken together, fPUC is rare. Locally advanced fPUC is even more rare. CHT rates in fPUC have increased over time in accordance with guideline recommendations. The effect of CHT independently predicts lower CSM. This observation validates guideline recommendation for use of CHT in fPUC patients based on strict statistical testing with proven independent predictor status. Its effect is equally pronounced in all three histological subgroups. However, due to sample size limitations, the independent predictor status for lower CSM could only be validated in the SCC subgroup. These observations strongly suggest that CHT exposure may improve survival in a statistically significant, but more importantly, in a clinically meaningful fashion in locally advanced fPUC.

Our study has several limitations due to the observational design and retrospective nature of the SEER database. Unfortunately, due to the rarity of fPUC, larger sample sizes than the ones recorded in the current study may not be expected. This sample size-/and power limitation will invariably undermine future studies that may be undertaken based on multi-institutional or other population databases. The National Cancer Database, that offers a larger pool of patients, may not provide meaningful answers when CHT is examined in the setting of locally advanced fPUC due to absence of CSM specific data. Finally, we could not address other patient’s variables that would have been used in ideal circumstances, such detailed as organ function, comorbidities or specific CHT eligibility criteria [18], since these were not reported in the SEER database.

## Conclusion

In fPUC patients, CHT use independently predicts substantially lower CSM. This effect also applies to SCC patients. The same relationship between CHT status and CSM is also operational in UC and ADK subgroups, but limited power undermined confirmation of its’ statistical significance.

## Data Availability

No datasets were generated or analysed during the current study.
